# Chronic Steroid Use Causing Spinal Epidural Lipomatosis

**DOI:** 10.7759/cureus.21945

**Published:** 2022-02-05

**Authors:** Dina Alnabwani, Nagapratap Ganta, Ryan Babayev, Vraj Patel, Viraj Shah, Pramil Cheriyath

**Affiliations:** 1 Internal Medicine, Hackensack Meridian Ocean University Medical Center, Brick, USA; 2 Internal Medicine, Rajarshee Chhatrapati Shahu Maharaj Government Medical College, Kolhapur, IND

**Keywords:** disc space narrowing, chronic steroid use, laminectomy, spinal epidural lipmatosis, steroid

## Abstract

Chronic steroid use causes aberrant fat deposition in the epidural space, which may in rare cases result in spinal epidural lipomatosis (SEL). We discuss the case of a 79-year-old female who had been on steroids for a long time, initially for polymyalgia rheumatica (PMR), then for adrenal insufficiency. Her dose was raised with a few steroid stress doses to control the flare of adrenal insufficiency. The patient presented with complaints of intractable lumbosacral pain and was subsequently diagnosed with SEL and foraminal and spinal canal stenosis based on magnetic resonance imaging of the lumbar spine. She successfully underwent laminectomy.

## Introduction

The abnormal build-up of fat in the epidural space of the vertebral canal is known as spinal epidural lipomatosis (SEL). This pathology is linked to patients who are on chronic corticosteroid medication (e.g., transplant, systemic lupus erythematosus, dermatomyositis, rheumatoid arthritis, and chronic obstructive pulmonary disease patients) [1]. Oral steroid intake on an average daily dose of 30-100 mg/day over 5-11 years can cause aberrant fat deposition in odd areas and the development of clinical symptoms. Some cases even showed these side effects with only short-term intravenous steroid use as well. Excessive endogenous glucocorticoid production linked with endocrinopathies like Cushing's syndrome is known to cause SEL. Obesity has been identified as another etiological factor, since endocrine dysfunction, metabolic abnormalities, and abnormal fat deposition are all linked. The most prevalent site of involvement is the thoracic spine, followed by the lumbosacral area [2]. The examination findings differ depending on whether the SEL is thoracic or lumbar (i.e., spinal cord, conus medullaris, and cauda equina). Magnetic resonance imaging is the preferred diagnostic modality to test for SEL [1].

## Case presentation

A 79-year-old female presented to the emergency department (ED) with a chief complaint of severe low back pain with radiation to the right leg, which started four weeks ago. She had a pre-existing problem of low back pain and spinal stenosis which started six months ago, but this pain increased and became unbearable. The pain was located in the left lumbosacral region with radiation down the right leg and occasionally down the left leg. She described her pain as stabbing in nature, constant, and 10/10 in intensity. She denied fever, chills, headache, nausea, vomiting, constipation, diarrhea, dysuria, frequency, and urgency. She had a blood pressure of 134/60 mmHg, heart rate of 94 bpm, respiratory rate of 18 per min, temperature of 36.2℃, SpO2 of 95% on room air, and body mass index of 30.3 kg/m^2^. Upon laboratory testing, she had a sodium of 131 mmol/L, potassium of 5.3 mmol/L, blood glucose of 123 mg/dL, creatinine of 1.42 mg/dL, and an anion gap of 10 mmol/L.

When the patient’s complaints initially began one month ago, she underwent a CT scan of the lumbar spine, which showed moderately severe degenerative joint disease, Grade 1 anterolisthesis of L4 on L5, multilevel disc space narrowing, and significant bilateral cyst and neural foraminal narrowing at L4-5. The patient's pain was managed, and she was discharged. 

The patient was given intravenous acetaminophen and diazepam in the ED, then was discharged on gabapentin and opioids. She had a medical history of coronary artery disease with stents, transcatheter aortic valve replacement, congestive heart failure, hypertension (HTN), polymyalgia rheumatica (PMR), chronic obstructive pulmonary disease, and anxiety. She has been fully vaccinated against COVID-19. 

She started taking prednisone 4 mg/day in September 2019 after being diagnosed with PMR. In September 2020, she was diagnosed with adrenal insufficiency, treated with a stress dose, then discharged with prednisone 7.5 mg/day until November 2020. This dose was later tapered over to 5 mg/day over nine weeks, which was continued until she was admitted with the complaint of back pain in late July 2021. She had also been on a pulse steroid treatment course with high-dose prednisone, which was then tapered to the 5 mg she normally takes for adrenal insufficiency. 

**Figure 1:**
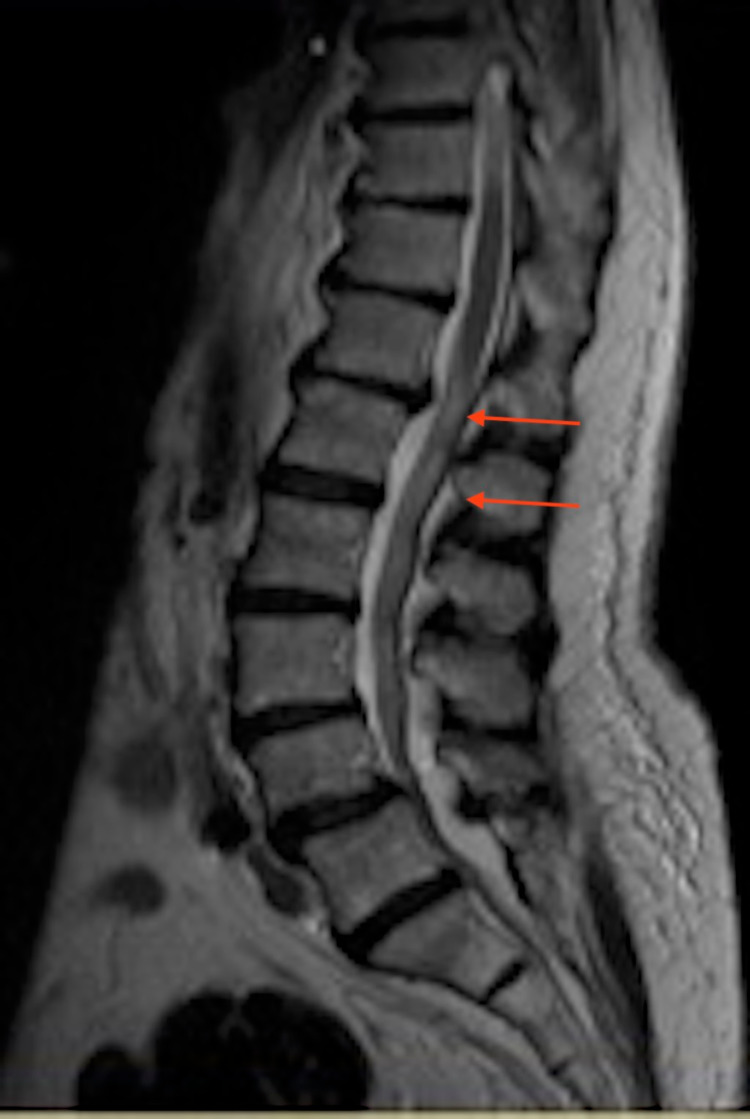
MRI lumbar spine showing arthritis and degenerative disc disease with epidural lipomatosis causing multilevel neural foraminal and spinal canal stenosis most pronounced at L4-L5 and L5-S1 (red arrows)

CT scan without contrast revealed Grade 1 anterolisthesis of L4 on L5, multilevel disc space narrowing (felt to be chronic in nature), bilateral lateral cysts, and neural foraminal narrowing at L4-5 secondary to hypertrophic changes. MRI of the lumbar spine showed arthritis and degenerative disc disease with SEL causing multilevel neural foraminal and spinal canal stenosis, most pronounced at L4-L5 and L5-S1 (Figure 1). The patient underwent surgery and laminectomy L4 with B partial medial facetectomies, laminectomy L5 with B partial medial facetectomies, laminectomy S1, posterior spinal fusion L45, and local bone graft. She was discharged after three days to a rehabilitation facility. 

Within a few days after discharge, the patient came to the emergency room with a complaint of seizure and possible loss of pulses from the rehabilitation facility. En route, the patient lost her pulses, and cardiopulmonary resuscitation (CPR) was started. She was admitted to inpatient hospice, but unfortunately, the patient passed away within a few days after admission.

## Discussion

SEL is a rare and unique pathological expansion of adipose tissue in the epidural area. It is most likely related to dural adipose tissue enlargement, which can constrict the spinal canal and induce cord compression if severe. This is most commonly seen in patients on exogenous steroid medication, although this can also occur in those with endocrinopathies, endogenous corticosteroid overproduction, obesity, or idiopathic disease [3]. SEL is an uncommon but well-documented side effect of long-term corticosteroid treatment, which was originally reported in 1975 in a young patient who received corticosteroids following a kidney transplant [4].

Compression of the spinal cord or nerve roots can cause gradual neurological impairments, even if symptomatic SEL is uncommon. This disorder appears to affect younger men more than women [5]. In a study of 104 cases of SEL, Fogel et al. discovered that most reported cases of SEL related to exogenous steroid administration were linked to long-term steroid use, with only three cases linked to repeated epidural steroid injections. Idiopathic cases of SEL have also been described in the absence of hypercortisolemia, mainly in obese patients. The prevalence of SEL in the setting of numerous epidural steroid injections for radicular pain has yet to be thoroughly studied. [5].

Adipose tissue generally accumulates in the thoracic spine, which is posterior to the spinal cord. The symptoms of SEL vary based on where damaged neural tissues are located. Compression at the thoracic level causes myelopathy, whereas compression at the lumbar level has radicular consequences [6]. Among the 104 cases studied by Fogel et al., about 46% involved only thoracic levels, 44% involved only lumbosacral levels, and the rest had lipomatosis in both locations. There were four cases that involved cervical levels, but none of them involved this area only [7].

SEL usually has an insidious and slow progression. Symptoms vary and may begin with vague back pain before progressing to lower extremity weakness and sensory loss. The neurological symptoms depend on the involved level of the spinal canal and neuronal structures (i.e., spinal cord, conus medullaris, or cauda equina). Thoracic involvement (58% to 61% of cases) can lead to progressive myelopathy, and generalized motor and sensory deficits can occur in the presence of spinal cord compression [7]. Lumbar involvement (39% to 42% of cases) can lead to radiculopathy with localized symptomatology, possibly due to the compression of specific nerve roots [6]. Incontinence of the bowel and bladder is uncommon. Further disease development is anticipated to have an equal impact on the thoracic and lumbar spinal areas [3].

MRI is the diagnostic modality of choice due to its superior and selective ability to discriminate fat content during imaging. T1-weighted pictures of adipose tissue are generally hyperintense, but water-filled portions of tissues can present similarly [3]. Circumferential compression of the thecal sac, also known as the “Y-sing,” is pathognomonic in lumbar axial imaging. Furthermore, epidural adipose tissue thicker than 7 mm is a diagnostic criterion for SEL [7]. In our patient, lumbar spine MRI revealed arthritis and degenerative disc disease with SEL causing multilevel neural foraminal and spinal canal stenosis, most pronounced at L4-L5 and L5-S1. 

Treatment options range from conservative therapy to spinal surgical surgery, depending on the severity of neurological impairments. Two of the more conservative alternatives include weaning off steroid therapy and losing weight [3]. Some patients, however, are on steroid medication for chronic diseases and may not be able to stop taking it. Notably, in the absence of exogenous steroid therapy, an evaluation for suspected endogenous corticosteroid overproduction is required. In many patients, decompressive laminectomy and adipose tissue excision have been beneficial surgical options. This operation relieves the compression by removing the lamina portion of the vertebrae at the level of epidural lipomatosis. After surgery, most patients achieve improvement or eradication of their neurological symptoms [3].

The patient underwent surgery and laminectomy L4 with B partial medial facetectomies, laminectomy L5 with B partial medial facetectomies, laminectomy S1, posterior spinal fusion L45, and local bone graft. She was discharged after three days to a rehabilitation facility. 

## Conclusions

SEL is an uncommon disorder in which excess adipose tissue accumulates in the spinal canal, causing symptoms of neurologic compression. This can occur in obese people and is an uncommon side effect of long-term steroid therapy. The diagnosis is solely based on imaging, specifically with MRI. Physicians should seek to treat the disorder's underlying cause, with surgical intervention reserved for individuals who have acute and severe symptoms or have failed to respond to conservative treatment. Further research is needed to better understand the related diseases to make an early, accurate diagnosis and plan adequate management.
